# Hypothyroidism and Mandibular Cortical Alterations Evaluated on Panoramic Radiography: A Cross-Sectional Study in Women

**DOI:** 10.3390/healthcare13192529

**Published:** 2025-10-07

**Authors:** Vicente Vera-Rodríguez, María Pedrera-Canal, Olga Leal-Hernández, Juan Fabregat-Fernández, María Luz Canal-Macías, Fidel López-Espuela, Francisco M. García-Blazquez, Jose M. Moran, Raúl Roncero-Martín

**Affiliations:** 1 Metabolic Bone Diseases Research Group, Nursing and Occupational Therapy College, University of Extremadura, 10003 Cáceres, Spainjuanfabregat@unex.es (J.F.-F.);; 2 Nuclear Medicine Service, Hospital San Carlos Clínica, Facultad de Medicina, Universidad Complutense de Madrid, 28040 Madrid, Spain; 3 Nursing Department, University of Extremadura, 10003 Cáceres, Spain

**Keywords:** hypothyroidism, mandible, radiography, panoramic, bone density, osteoporosis, levothyroxine

## Abstract

**Highlights:**

**What are the main findings?**

- In adult women treated for primary hypothyroidism, panoramic mandibular cortical markers (MCI, PMI, and MCW) did not significantly differ from those in euthyroid controls after adjusting for age. While the left MCW initially appeared higher, this difference was found to be statistically insignificant after age adjustment, and no relevant differences were observed in the right MCW.
- This study found no significant differences in mandibular cortical indices (MCI, PMI, MCW) between women treated for primary hypothyroidism and euthyroid controls after age adjustment. Beyond the specific findings, the value of this work lies in its integration of endocrinological and maxillofacial perspectives, demonstrating how systemic endocrine conditions may have radiographic correlates in dental imaging.

**What is the implication of the main finding?**

- These findings suggest that routinely derived panoramic indices (MCI, PMI, MCW) have limited discriminatory value for detecting mandibular cortical alterations in women treated for primary hypothyroidism. Future studies should incorporate longitudinal, multicentre designs with rigorously controlled confounders (such as age, menopausal status, BMD, and LT4 treatment parameters) to clarify potential subtle effects and assess their clinical relevance.
- While panoramic indices appear to have limited utility in detecting mandibular cortical alterations in treated hypothyroid patients, the interdisciplinary approach of this study reinforces the potential of dental radiographs as a window into systemic health. Highlighting this intersection strengthens the clinical relevance of the findings and supports broader collaboration between medical and dental specialties.

**Abstract:**

Background/Objectives: Thyroid hormone deficiency may impair bone metabolism, but its mandibular impact remains uncertain. We aimed to compare the prevalence of altered Mandibular Cortical Index (MCI; C2–C3) and Panoramic Mandibular Index (PMI) on digital panoramic radiographs in adult women with primary hypothyroidism versus euthyroid controls, considering age and key clinical covariates. To our knowledge, this is the first study out of Spain addressing this question. Methods: This is a cross-sectional study (September 2021–June 2024) of 179 white women recruited at a university clinic. Cases were on L-thyroxine for ≥6 months, with TSH > 4.5 mIU/L and normal FT4; controls were euthyroid and untreated. Demographics, reproductive history, and BMI were recorded. Panoramic radiographs (Ratograph EVO 3D; 72 kV, 6 mA, 14.4 s) were analysed; MCI was graded (Klemetti C1–C3) distal to the mental foramen; PMI and mandibular cortical width (MCW) were measured bilaterally. Results: Women with hypothyroidism showed higher BMI and a greater number of years since menopause; age was slightly higher, but the difference was not significant. MCI distribution did not differ between groups (C3 and C2–C3, both *p* > 0.45). PMI (left/right) was similar (*p* = 0.253/0.160). Left MCW was higher in hypothyroidism in a crude analysis (4.86 ± 0.98 vs. 4.46 ± 0.94 mm; *p* = 0.039), but lost significance after age adjustment (adjusted *p* = 0.191); right MCW showed no differences. Total tooth loss tended to be higher (*p* = 0.055) without conclusive evidence. Conclusions: In this cohort, primary hypothyroidism was not associated with a differential mandibular cortical pattern by MCI or PMI; the crude MCW difference was explained by age. These Spain-based data refine heterogeneous prior findings and indicate that, in women treated for hypothyroidism, mandibular cortical metrics largely resemble those of their euthyroid peers. Longitudinal and multicentre studies are warranted to clarify trajectories and enhance generalisability.

## 1. Introduction

Thyroid hormones are essential for skeletal development and maintaining bone health and balance in adults [1,2,3,4,5]. An imbalance, such as hypothyroidism (a condition where the thyroid gland does not produce enough hormones) [6], affects numerous tissues and organs [1]. This condition, as one of the most common endocrinopathies, has a prevalence between 3.8% and 4.6% of the general population and is 5 to 10 times more common in women [1,3,7]. Hypothyroidism disrupts bone metabolism, dampening the activity of both bone-forming cells (osteoblasts) and bone-resorbing cells (osteoclasts) [2,8,9].

The standard treatment for hypothyroidism is long-term hormone replacement therapy with L-thyroxine (LT4), a synthetic thyroid hormone [1,7,10]. However, prolonged use of LT4 has been identified as a significant risk factor for osteoporosis, as it can increase osteoclast activation [7]. In fact, despite adequate hormone therapy, it has been documented that between 20% and 50% of patients with primary hypothyroidism have osteoporosis and osteopenia [2]. Osteoporosis significantly affects patients’ quality of life by deteriorating the bone microarchitecture, reducing bone mass, and increasing the risk of fractures [1,2,11].

Although it has long been known that LT4 increases the risk of fractures and decreases bone mineral density in regions such as the forearm, hip, femoral head, and vertebrae, there is conflicting data in the literature regarding its specific effect on the maxillary bones [1]. This knowledge gap pertaining to a part of the skeleton which is crucial to daily functioning and quality of life justifies in-depth research into the impact of hypothyroidism and its treatment on oral health.

To address this issue, panoramic X-rays are utilised—an excellent tool that is routinely employed and readily accessible in dentistry [12,13,14,15,16]. These X-rays are less expensive and involve less radiation exposure than other, more advanced imaging modalities, such as DEXA or tomography [1,3]. From these X-rays, radiomorphometric indices can be extracted, such as the Mandibular Cortical Index (MCI), the Panoramic Mandibular Index (PMI), the Mental Index (MI), the Antigonial Index (AGI), and the Gonial Index (GI), which are valuable indicators of bone mineral density (BMD) [2,12,17]. The MCI is particularly practical because it can be easily determined [18]. These indices can ultimately help in the early identification of individuals with low BMD or those who are at risk of osteoporosis [3].

However, the findings in the literature are varied, contributing to persistent ambiguity regarding bone metabolism alterations in patients with hypothyroidism [1,2,3]. In premenopausal women undergoing levothyroxine therapy, MCI differed from healthy controls (higher frequency of C2 in women undergoing treatment), suggesting a possible cortical impact without noticeable trabecular changes [1]. In contrast, a retrospective study of patients with hypothyroidism undergoing treatment found similar values for PMI and MCI (among others) compared to controls [2], while another series in adults with hypothyroidism observed subtle differences (different MCI distribution) that point to subtle cortico-mandibular changes [3]. In hypothyroidism, the potential impact is concentrated in the cortex, where MCI emerges as a highly sensitive marker for detecting subtle alterations [19]. Beyond bone loss, hypothyroid patients receiving thyroxine replacement therapy have shown greater periodontal destruction compared to controls, even without apparent radiomorphometric differences, suggesting an additional inflammatory component relevant to oral health [20]. Recent comparative studies on endocrinopathies indicate that, unlike hyperthyroidism, research on cortical parameters, such as MCI, must be improved in hypothyroidism, particularly in female populations [21].

Given the potential cortical implications of hypothyroidism and the limitations of existing data (especially in Southern European populations), the aim of this study was the evaluation of mandibular cortical alterations in women with primary hypothyroidism using panoramic radiographic indices. These include the MCI, PMI, and MCW. This study was conducted in Spain, where no prior analyses of this nature have been reported.

The central hypothesis is that women treated for primary hypothyroidism will exhibit differences in mandibular cortical indices compared to euthyroid controls, even after age adjustment. By clarifying whether these radiographic markers capture meaningful bone alterations in this context, we also seek to assess the utility of panoramic imaging as a low-cost, opportunistic screening tool for systemic skeletal conditions.

## 2. Materials and Methods

### 2.1. Study Sample

This cross-sectional study was conducted between September 2021 and June 2024. It included 179 white women treated at the Bone Metabolic Diseases Research Group (GIEMO) of the Faculty of Nursing and Occupational Therapy of Cáceres, University of Extremadura (Spain). The characteristics of the sample were previously reported [22]. The final sample consisted of 179 women: 130 with a confirmed diagnosis of primary hypothyroidism (under stable L-T4 treatment) and 49 euthyroid controls. Potential confounding variables (such as age, menopausal status, supplementation use, and comorbidities) were documented and considered in the statistical analyses.

The study women were ≥18 years of age and with TSH > 4.5 mIU/L and serum-free T4 within the normal range (0.8–1.2 ng/dL) [23]. The case group comprised those undergoing thyroid hormone replacement therapy for ≥6 months, while the control group was without such therapy. In all hypothyroid patients, LT4 dosage was regularly adjusted to maintain TSH and FT4 within reference ranges, ensuring stable replacement therapy at the time of inclusion. The exclusion criteria comprised clinical osteoporosis and routine medication that interfered with vitamin D or bone metabolism. All participants were residents of the urban area within the Cáceres health district, Spain. Most participants were married, had children, and belonged to a middle socioeconomic stratum. None reported dietary limitations, neurological disorders, or physical impairments, and their medical records revealed no history of low-impact fractures. Prior to inclusion in this study, each woman underwent a comprehensive medical history review and a physical examination.

Regarding pharmacological profiles, 14.5% of the women reported calcium supplementation and 11.2% took vitamin D. However, no participants were using antiresorptive agents or other medications known to significantly alter bone metabolism, such as corticosteroids, oral anticoagulants, or antipsychotic drugs. The only exception was levothyroxine (L-T4), used in the hypothyroid group and, in some cases, hormone replacement therapy. All women led generally active lifestyles, though none engaged in regular sports. Alcohol consumption was sporadic and did not exceed 100 mL per day. Tobacco use was low, with 17.3% of participants identified as current smokers.

All participants signed written informed consent forms, and the Ethics Committee of the University of Extremadura approved the protocol in December 2020 (ref. 192/2020).

### 2.2. Study Variables

The age, age at menarche, gonadal status, and years since menopause were recorded for each participant. Postmenopause was defined as the absence of menstruation for ≥12 months. Anthropometric measurements were obtained according to the recommendations of the Spanish Society for the Study of Obesity [24]; body mass index (BMI) was calculated using height and weight.

### 2.3. Mandibular Cortical Assessment

A qualified operator obtained mandibular orthopantomograms with a Ratograph EVO 3D (Villa Sistemi Medicali, Milan, Italy) set to 72 kV, 6 mA, and 14.4 s exposure. The images were stored in JPEG format (1536 × 2573 pixels) and processed with MATLAB R2018b.

The MCI was graded according to the Klemetti index [25] by evaluating the cortex distal to the mental foramen: C1, smooth and well-defined endosteal margin on both sides; C2, presence of semilunar defects (lacunar resorption) and endosteal cortical remnants on one or both sides; C3, porous cortex with marked accumulation of endosteal remnants.

### 2.4. Bone Mineral Density

The BMD of the trochanter, lumbar spine L2–L4, and femoral neck was assessed using DXA with a Norland XR-800 (Fort Atkinson, WI, USA), expressing the results as mineral mass per scanned area (g/cm^2^). According to WHO criteria [26], osteoporosis was diagnosed when the T-score was <−2. The T-score is the number of standard deviations separating the subject’s BMD from the mean of a young adult reference population.

### 2.5. Artificial Intelligence in Manuscript Refinement

Artificial intelligence tools (Grammarly and DeepL) were employed to refine the manuscript’s language, enhance clarity, and better articulate complex ideas.

### 2.6. Statistical Analysis

Continuous variables are described as mean (standard deviation) and categorical variables as n (%). Student’s *t*-test was used for comparisons between groups (if assumptions of normality and homoscedasticity were met); otherwise, the Mann–Whitney U test was used. Normality was assessed using the Shapiro–Wilk test, and homoscedasticity was evaluated using Levene’s test. Where appropriate, age-adjusted *p*-values were calculated. All tests were two-tailed with α = 0.05. Analyses were performed using JASP (JASP Team, Amsterdam, The Netherlands).

## 3. Results

In the hypothyroid status comparison (Table 1), women with hypothyroidism had a higher BMI and a greater number of years since menopause. Age was higher in the hypothyroidism group, but this was insignificant. No differences were observed between groups in densitometric distribution (normal/osteopenia/osteoporosis) or gonadal status.

In cortical indices (Table 2), left MCW was higher in the hypothyroidism group; however, the difference was attenuated after adjusting for age (adjusted *p* = 0.191). Right MCW showed no statistically significant differences. Left PMI and right PMI were similar between groups. There was a trend toward greater total tooth loss in hypothyroidism (borderline *p*-value), with no conclusive evidence of difference. In the Klemetti index, neither category C3 nor the C2–C3 group differed between groups.

In the subgroup of premenopausal women (n = 23), no statistically significant differences were observed between hypothyroid (n = 5) and control participants (n = 18) for any of the radiomorphometric indices or dental variables assessed. The mean left MCW was higher in hypothyroid women (5.45 ± 0.82 mm) compared to controls (4.19 ± 1.13 mm), but the difference was statistically insignificant (t = −2.026, *p* = 0.064). Similarly, no significant group differences were found for right MCW (5.2 ± 1.4 vs. 5.0 ± 0.8 mm; *p* = 0.690), left PMI (0.37 ± 0.06 vs. 0.29 ± 0.08; *p* = 0.114), right PMI (0.33 ± 0.08 vs. 0.35 ± 0.06; *p* = 0.572), or total teeth lost (1.6 ± 3.6 vs. 0.8 ± 2.0; *p* = 0.778). In postmenopausal women (n = 156), no statistically significant differences were observed between hypothyroid (n = 44) and euthyroid controls (n = 112) for any radiomorphometric or dental variable. Mean left MCW was 4.79 ± 0.99 (n = 31) in hypothyroid participants vs. 4.51 ± 0.92 (n = 78) in controls (*p* = 0.156); right MCW was 5.36 ± 0.98 (n = 44) vs. 5.04 ± 0.99 (n = 112; *p* = 0.068). Left PMI was 0.324 ± 0.084 (n = 31) vs. 0.314 ± 0.075 (n = 78; *p* = 0.545), and right PMI 0.378 ± 0.089 (n = 44) vs. 0.355 ± 0.081 (n = 112; *p* = 0.118). Total teeth lost averaged 6.11 ± 8.34 (n = 44) in hypothyroid women and 4.28 ± 7.55 (n = 112) in controls (*p* = 0.187).

When stratifying the analysis by gonadal status, no statistically significant associations were observed between hypothyroidism and mandibular cortical morphology, whether assessed by Klemetti C3 or C2/C3 categories. Among premenopausal women (n = 23), one of five hypothyroid participants presented C3 morphology compared with none of the 18 controls (χ^2^ = 3.764, *p* = 0.052), while C2/C3 morphology was observed in 2 of 5 hypothyroid participants and in 12 of 18 controls (χ^2^ = 1.168, *p* = 0.280). In postmenopausal women (n = 156), C3 morphology was present in 10 of 44 hypothyroid participants and in 23 of 112 controls (χ^2^ = 0.091, *p* = 0.763), whereas C2/C3 morphology was observed in 35 of 44 hypothyroid participants and in 82 of 112 controls (χ^2^ = 0.675, *p* = 0.411).

## 4. Discussion

In this cohort of women, comparison by thyroid status revealed no apparent differences in key radiomorphometric markers of the mandibular cortex (MCI (categories C3 and C2–C3) and PMI) and an initially higher left MCW in hypothyroidism that attenuated after adjusting for age, with no changes on the right side. This pattern places our findings within the range of conservative results described in the literature, where several studies on hypothyroid patients undergoing treatment did not detect significant differences in MCI/PMI/MCW compared to controls, nor did they report subtle variations dependent on age and other confounders, such as menopausal status or exposure to LT4. Taken together, our results are compatible with two non-exclusive explanations: (i) effective replacement therapy may attenuate cortical differences attributable to hypothyroidism under real-world conditions, and (ii) methodological constraints of panoramic imaging can introduce non-differential measurement error that biases small effects towards the null. In addition, although we documented and adjusted for major confounders (e.g., age as a continuous covariate), residual confounding from unmeasured local factors (periodontal burden and tooth loss patterns) cannot be excluded.

Our observations are consistent with those of Günen Yilmaz and Bayrak (2023) and Rahangdale and Galgali (2018) [14,20], who found no statistically significant differences in average MCW between patients with hypothyroidism (treated with LT4) and the control groups. Although the average MCW in the hypothyroid group was slightly lower in some studies, this difference was insignificant. These results contrast with those of Gulec et al. (2023), who reported statistically significant MCI scores between patients using LT4 long-term and control subjects (*p* = 0.016). The C2 score (moderately resorbed cortex) was higher in the case (36.6%) than in the control group (15.5%), while C1 (normal cortex) was more frequent in controls (83.1%). According to the authors, this indicates that the use of LT4 could affect the mandibular cortical layer’s direction of resorption [1]. Recently, Nair et al. (2025) also reported a significant difference in mandibular cortical index (MCI) levels between patients with hypothyroidism and controls (*p* = 0.001). Interestingly, most cases with hypothyroidism had a C1-level MCI (55.3%), suggesting uniform and sharp endosteal margins without much resorption. In comparison, the control group had a higher percentage of C2 patterns (74.0%), indicating semilunar defects or lacunar erosions. This observation is contrary to what would be expected if hypothyroidism caused greater resorption [3]. Such inconsistencies across studies may reflect differences in population structure (age/menopausal mix), treatment stability (dose and duration of LT4), supplementation profiles (in our sample, 14.5% received calcium and 11.2% vitamin D), imaging protocols, and reader blinding; selective reporting and small-study effects may also contribute to apparent discordance in the literature.

In our study, we also observed no differences in PMI results, confirming the previous findings of Günen Yilmaz and Bayrak (2023), who observed no significant differences in PMI values between the hypothyroid group (treated with LT4) and the control group [14]; Rahangdale and Galgali (2018) also reported that PMI did not differ significantly between hypothyroid patients and controls [20]. Similarly, Nair et al. (2025) observed lower PMI values in the hypothyroid group, but this difference was not statistically significant [3]. Consistent with these patterns, our stratified analyses by gonadal status (premenopausal/postmenopausal) did not identify significant differences in Klemetti categories or continuous indices, supporting a cautious interpretation that panoramic indices have limited discriminatory capacity in treated hypothyroid women at the group level.

The wide variability observed between the different studies collected in the literature can be attributed to the differences between the study populations. Some studies specifically excluded postmenopausal women to control for the variability in bone density induced by hormonal changes associated with menopause. This strict control of the population may have allowed for the detection of a significant difference in BMD (higher C2 in LT4 users), which could have been diluted in more diverse or postmenopausal populations [1]. Other studies included a wider age range and both genders, which may introduce more variability.

Nair et al., in their study that predominantly included middle-aged women and did not focus exclusively on premenopausal women, found a significant difference in MCI but with an unexpected pattern (more C1 in hypothyroid individuals), which could reflect the complexity of the effects of hypothyroidism in a more diverse population or the influence of unmeasured factors [3]. The duration of hypothyroidism and LT4 treatment is also a relevant factor. While some studies in patients treated with LT4 found no significant differences in several indices (suggesting possible bone stabilisation with therapy) [14], others did observe possible resorptive effects of long-term LT4 on the mandibular cortex [1]. Our data cannot adjudicate definitively between these mechanisms; rather, they suggest that under routine clinical management, any cortical signal detectable on panoramic imaging (if present) is likely modest and sensitive to age and treatment-related context.

In our study, we acknowledge the following limitations: Our cross-sectional, clinically based design with a non-probabilistic sample (from a single centre) prevents us from establishing temporality and limits external validity (specifically for Spanish white women), in addition to susceptibility to selection bias. The design also restricts the establishment of causality or evaluation of longitudinal changes in mandibular cortical morphology. To clarify how thyroid disorders affect the mandibular bone structure and optimise their detection, future research must resolve the limitations identified in current studies and significantly expand the methodological scope. We must transcend the cross-sectional and retrospective design prevalent in the current literature. We need prospective, longitudinal studies that follow patients over time. This will allow us to observe the evolution in mandibular bone architecture from before diagnosis or treatment initiation and assess the real impact of therapy over an extended period. The heterogeneity and size of populations are another critical area of concern. The unequal sample sizes between the hypothyroid and control groups may introduce statistical imbalance. While this reflects real-world clinical prevalence, it may influence the robustness of group comparisons. We sought to mitigate this limitation through appropriate statistical adjustments, particularly for age (a known confounder in bone-related outcomes). Nevertheless, the findings should be interpreted with caution, and replication in larger, balanced cohorts is recommended. Power limitations in the premenopausal subgroup (n = 5 hypothyroid) also increase the risk of type II error.

Future research should include larger samples to increase statistical power and the ability to detect significant differences that may currently be overlooked. This includes consideration of hormone levels and medication dosages, menopausal status, and control of comorbidities and lifestyle habits. In addition, harmonised imaging protocols with blinded, duplicate readings and validation against higher-resolution modalities (e.g., CBCT or DEXA) are needed to quantify measurement error and to test whether panoramic indices can serve as reliable triage tools in clinical workflows.

For dental practitioners, panoramic radiographs should not be used as standalone diagnostics of systemic skeletal status in treated hypothyroid women; rather, conspicuous cortical thinning/porosity (e.g., Klemetti C3) should prompt documentation of endocrine history (hypothyroidism, LT4 stability, calcium/vitamin D, bone-active drugs) and, where risk factors co-occur (age, prior low-trauma fracture, periodontal loss), communication with primary care/endocrinology for formal osteoporosis assessment. For medical teams, routine dental panoramics can be considered opportunistic signals to be integrated with established risk tools (e.g., clinical risk factors, BMD testing), rather than substitutes for them. This coordinated approach can be expected to maximise patient benefit while avoiding over-interpretation of 2D indices.

## 5. Conclusions

In this cohort of women, primary hypothyroidism did not show a differential pattern of mandibular cortical thinning according to MCI/PMI; the greater left MCW observed in the crude analysis was diluted after adjustment for age, suggesting minor differences. However, due to the observational nature of this study and the inherent limitations of panoramic imaging, these results should be interpreted with caution and not as definitive evidence of the absence of effect. These findings (the first reported in Spain) provide local data that contribute to contextualising the previously reported heterogeneity in the literature. It remains plausible that mandibular cortical alterations could be influenced by hypothyroidism in interaction with other systemic, local, or therapeutic factors not fully captured in this study.

Looking ahead, it would be beneficial to address this question through prospective longitudinal cohorts that enable individual trajectories to be followed, multicentre studies that enhance generalisability, and matched case–control designs (including the nested variant) for more nuanced contrasts. Additionally, pragmatic trials of diagnostic strategies could assess the actual contribution of radiographic indices to clinical management. From a clinical perspective, while the diagnostic value of panoramic indices appears limited in treated hypothyroid women, their inclusion in routine dental imaging offers a non-invasive, opportunistic screening tool. Recognising systemic conditions that may subtly influence mandibular bone structure supports integrated care and may prompt further endocrinological evaluation when warranted. This underscores the potential value of interdisciplinary awareness in daily clinical practice.

## Figures and Tables

**Table 1:** Baseline characteristics by hypothyroidism status.

Variable	Hypothyroidism: No (n = 49)	Hypothyroidism: Yes (n = 130)	*p*-Value
Age (years) †	57.68 (9.81)	60.92 (7.99)	0.098
BMI (kg/m^2^) ‡	26.28 (4.28)	28.30 (5.43)	0.010
Years since menopause §	9.46 (9.17)	12.88 (9.51)	0.014
Osteoporosis diagnosis			0.998
Normal	16 (32.7%)	16 (32.7%)	
Osteopenia	21 (42.9%)	21 (42.9%)	
Osteoporosis	12 (24.5%)	12 (24.5%)	
Gonadal status			0.516
Premenopausal	5 (10.2%)	18 (13.8%)	
Postmenopausal	44 (89.8%)	112 (86.2%)	

Table 1 compares key demographic and clinical variables between women with and without a diagnosis of primary hypothyroidism. Continuous variables (age, BMI, and years since menopause) are presented as means with standard deviations; categorical variables (osteoporosis diagnosis and gonadal status) are expressed as frequencies and percentages. Sample sizes vary by variable due to missing data: † Age—No n = 49; Yes n = 130. ‡ BMI—No n = 49; Yes n = 128. § Years since menopause—No n = 49; Yes n = 129. *p*-values for continuous variables were obtained using Student’s *t*-test or Mann–Whitney U test, as appropriate; categorical variables were compared with the χ^2^ test.

**Table 2:** Mandibular radiomorphometric indices by hypothyroidism status.

Variable	Hypothyroidism: No (n = 49)	Hypothyroidism: Yes (n = 130)	Effect Size	95% CI for Effect Size	*p*-Value	Adjusted *p*-Value
Left MCW (mm) †	4.46 (0.94)	4.86 (0.98)	−0.417	−0.811; −0.22	0.039	0.191
Right MCW (mm) ‡	5.30 (0.96)	5.34 (1.013)	−0.319	−0.649; 0.011	0.058	
Left PMI §	0.35 (0.08)	0.36 (0.08)	−0.229	−0.621; 0.163	0.253	
Right PMI ¶	0.35 (0.07)	0.37 (0.08)	−0.236	−0.566; 0.093	0.160	
Total teeth lost ‖	3.80 (7.10)	5.60 (8.08)	−0.168	−0.345; 0.021	0.055	
Klemetti index						
C3	23 (17.7%)	11 (22.4%)			0.469	
C2 or C3	94 (72.3%)	37 (75.5%)			0.666	

Table 2 presents a comparison of mandibular cortical indices and dental status between women with and without hypothyroidism. Mean values and standard deviations are provided for continuous variables (MCW, PMI, and total tooth loss), along with both unadjusted and age-adjusted *p*-values where applicable. The Klemetti index categories are shown as frequencies and percentages. In addition, effect sizes and their 95% confidence intervals are reported to complement the *p*-values and provide a clearer estimate of the magnitude and precision of the observed differences. Data are mean (SD) or n (%). Sample sizes vary by variable due to missing data: † Left MCW—No n = 89; Yes n = 35. ‡ Right MCW—No n = 130; Yes n = 49. § Left PMI—No n = 130; Yes n = 49. ¶ Right PMI—No n = 130; Yes n = 49. ‖ Total teeth lost—No n = 49; Yes n = 130. *p*-values for continuous variables were obtained using Student’s *t*-test or Mann–Whitney U test, as appropriate; categorical variables were compared with the χ^2^ test. Age-adjusted *p*-values are shown when available.

## Data Availability

The raw data supporting the conclusions of this article will be made available by the authors on request.
